# Differentiated Care Preferences of Stable Patients on Antiretroviral Therapy in Zambia: A Discrete Choice Experiment

**DOI:** 10.1097/QAI.0000000000002070

**Published:** 2019-04-19

**Authors:** Ingrid Eshun-Wilson, Mpande Mukumbwa-Mwenechanya, Hae-Young Kim, Arianna Zannolini, Chanda P. Mwamba, David Dowdy, Estella Kalunkumya, Mwansa Lumpa, Laura K. Beres, Monika Roy, Anjali Sharma, Steph M. Topp, Dave V. Glidden, Nancy Padian, Peter Ehrenkranz, Izukanji Sikazwe, Charles B. Holmes, Carolyn Bolton-Moore, Elvin H. Geng

**Affiliations:** a University of California, San Francisco, San Francisco, CA;; b Centre for Infectious Disease Research in Zambia, Lusaka, Zambia;; c Africa Health Research Institute, Durban, South Africa;; d United Kingdom Department for International Development, Dar Es Salaam office, Tanzania;; e Johns Hopkins University, Baltimore, MD;; f James Cook University, Townsville, Australia;; g University of California, Berkeley, Berkeley, CA;; h Bill and Melinda Gates Foundation, Seattle, WA;; i Georgetown University, Washington, DC; and; j University of Alabama at Birmingham, Birmingham, AL.

**Keywords:** differentiated care, HIV, discrete choice, antiretroviral therapy, preference

## Abstract

Although differentiated service delivery (DSD) models for stable patients on antiretroviral therapy (ART) offer a range of health systems innovations, their comparative desirability to patients remains unknown. We conducted a discrete choice experiment to quantify service attributes most desired by patients to inform model prioritization.

**Methods:**

Between July and December 2016, a sample of HIV-positive adults on ART at 12 clinics in Zambia were asked to choose between 2 hypothetical facilities that differed across 6 DSD attributes. We used mixed logit models to explore preferences, heterogeneity, and trade-offs.

**Results:**

Of 486 respondents, 59% were female and 85% resided in urban locations. Patients strongly preferred infrequent clinic visits [3- vs. 1-month visits: β (ie, relative utility) = 2.84; *P* < 0.001]. Milder preferences were observed for waiting time for ART pick-up (1 vs. 6 hours.; β = −0.67; *P* < 0.001) or provider (1 vs. 3 hours.; β = −0.41; *P* = 0.002); “buddy” ART collection (β = 0.84; *P* < 0.001); and ART pick-up location (clinic vs. community: β = 0.35; *P* = 0.028). Urban patients demonstrated a preference for collecting ART at a clinic (β = 1.32, *P* < 0.001), and although most rural patients preferred community ART pick-up (β = −0.74, *P* = 0.049), 40% of rural patients still preferred facility ART collection.

**Conclusions:**

Stable patients on ART primarily want to attend clinic infrequently, supporting a focus in Zambia on optimizing multimonth prescribing over other DSD features—particularly in urban areas. Substantial preference heterogeneity highlights the need for DSD models to be flexible, and accommodate both setting features and patient choice in their design.

## Abstract 2

Supplemental Digital Content is Available in the Text.

## INTRODUCTION

Differentiated service delivery (DSD) models for clinically stable HIV patients in Africa may be optimally effective when a particular model both fits with existing health systems and matches preferences of patients in a particular setting. At present, many promising DSD models exist, but their comparative desirability to patients remains incompletely understood. Facility-based individual models, which include multimonth prescribing and “fast tracking” primarily reduce clinical contact, and thus, opportunity costs (the cost to the patient of attending health services in terms of time and money) while requiring minimal changes to logistics, infrastructure, or human resources. Other models, which distribute antiretroviral therapy (ART) in the community include community-based health care worker-managed groups, client-managed groups, and out-facility individual models. Community ART distribution in these models is achieved through home, community venue, and mobile drug distribution points, and in some instances, the creation of patient groups (client-managed groups) that rotate individuals to pick-up ART for all at the facility and distribute in the community.^1^ These models may reduce geographical barriers to care but require equipment (eg, mobile vans), patient cooperation (for client-managed groups), or risk privacy in the community. At this juncture, some settings have reported slow enrollment into DSD models^2,3^ while others have concerning levels of drop-out.^4–6^ Given the large number of models which national agencies must choose from, data on the comparative desirability of DSD model features to patients can help national health agencies prioritize their use.

Research on DSD to date has largely focused on comparisons between a model and traditional, facility-based care with frequent visits, but has less frequently compared different DSD models with each other, in particular through soliciting patient preferences for different models. Experimental studies suggest^4,7–9^ that, in general, patients benefit from peer support and other model components, which reduce visit frequency and waiting time.^10,11^ But these studies do not address what services patients most want. For example, although early experience reported patient enthusiasm for client-managed groups, it remains unknown how much of this desire is driven by the social support from the groups as compared to the reduced opportunity costs of treatment afforded by membership in the group and reduced burden of clinic visits. Although qualitative studies suggest that client-managed groups work through both social support and opportunity costs,^10,11^ these desires are not quantified vis a vis another desired characteristic of health care. A quantitative measure of patient preferences could advance our understanding not only of what models work, but also what about them works, and thereby allow evidence-based design of differentiated care packages most appealing to patients, subgroups, and health care settings.^1^

In this article, we present findings from a discrete choice experiment (DCE) to assess the preferences of patients receiving HIV care in Zambia. A choice experiment can present several hypothetical scenarios of a health service to patients. By varying the service attribute levels (eg, 2 clinics with different waiting times and ART refill frequencies), such an experiment can demonstrate patient preferences and the amount of another attribute they would be willing to trade. DCEs are gaining recognition in HIV research and have been used to explore the patient preferences for HIV prevention interventions,^12^ HIV testing,^13–15^ and ART services.^16,17^ We used this approach in Zambia to determine what DSD features stable patients on ART most prefer.

## METHODS

### Population and Sampling

We nested this study within a general survey which formed part of a larger study evaluating the implementation of differentiated care models in Zambia. The survey was conducted across 3 provinces (Southern, Eastern, and Lusaka) and 12 Ministry of Health (MoH) health care facilities supported by Centre for Infectious Disease Research in Zambia (CIDRZ). Facilities included 5 rural (approximately 1000–2000 patients per facility) and 7 urban sites (more than 3000 patients per facility) that were providing standard care and no DSD interventions. A consecutive sampling technique was used to recruit a total sample size at each site that was proportional to the overall size of the clinic ranging from 7 to 207 patients (2.5% of total clinic population). Participants were eligible if they were 18 years or older, gave consent to participate in the survey and DCE, and if they were the only household member recruited. For the survey, both ART and non-ART patients were eligible. The DCE component was then offered to a randomly selected subset of ART participants. Randomization was determined through random number generation on an electronic tablet at the time of the survey (see Supplemental Digital Content 1, http://links.lww.com/QAI/B323).

### Selection of DSD Model Features for the Choice Experiment

We conducted a literature review and consulted local stakeholders to determine which DSD features (attributes) would be most relevant in the Zambian setting. Long waiting times, high-frequency clinic visits, and lack of support are frequently cited barriers to retention in HIV care,^10,18,19^ and these are some of the main health care features which DSD models aim to address by adjusting visit frequency, location of services, mode of deliver, and type of service. Current differentiated service packages for stable patients include (1) health care worker-managed groups, where groups meet within or outside health care facilities, have reduced clinical visit and ART pick-up frequency, and receive group adherence counseling sessions, (2) client-managed groups which mostly operate outside the facility, where ART collection occurs within the community facilitated by the group, there are infrequent clinical assessments at the health facility and group counseling, (3) facility-based individual models where patient ART refill visits are expedited and bypass clinic staff, and there are infrequent clinical assessments and group adherence counseling, and (4) out-of-facility individual models where ART or clinic consultations are provided outside of the health facility with individualized adherence support.^1^ Based on these models, we selected 6 DSD features that were most relevant to the Zambian setting and that were also formed part of the intervention arms of the larger trial (Table 1). At the time of the experiment, multimonth ART prescriptions were not available as part of standard care in Zambia. The DSD feature levels were determined according to guidance from local staff with regard to current care practices regarding frequency of clinic visits and waiting times, and what would be acceptable and understandable to the local population. We then piloted the DCE in 2 phases on 2 groups of 5–10 participants and amended attribute levels, phrasing of questions and training materials based on participant responses.

**TABLE 1.**
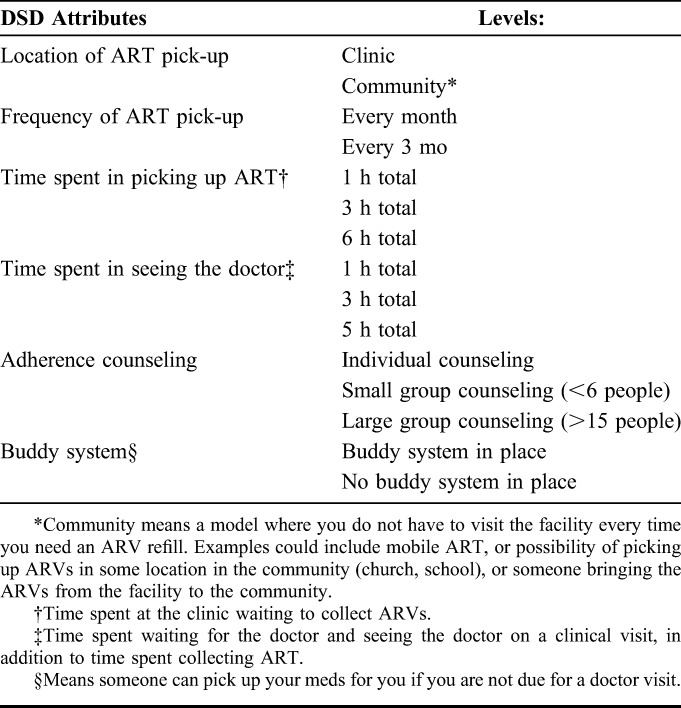
Differentiated Service Features (Attributes) Selected for DCE

### DCE Design

There was a total of 216 possible combinations of the DSD attributes and levels that were selected for the experiment (2 × 2 × 3 × 3 × 3 × 2), resulting in 23,220 total combinations [(216 × 215)/2] across 2 clinic scenarios, clinic A or clinic B. This number of combinations could not possibly be presented to patients; we therefore used a fractional factorial design to reduce the number of choice sets presented to an individual patient.^20^ Based on good practice guidelines, we considered that a maximum of 14 choice tasks would ensure response and statistical efficiency.^21^ We used STATA version 14 to design a statistically efficient experiment with a total of 14 choice sets in 2 blocks of 7 questions each. The design was based on a model with 9 estimable parameters and no interactions between attributes. The generated design was optimally D-efficient within the constraints provided. To further evaluate the overall design, we explored the correlation between attributes, which was low overall (<0.30) (see Supplemental Digital Content 2, http://links.lww.com/QAI/B323).

### Sample Size Determination

Estimation methods for sample size determination for DCEs vary and are dependent on the number of attributes, levels, heterogeneity of preferences, desired level of precision, interactions between attributes, number of choice tasks, and alternative scenarios presented in the experiment,^22^ and as a rule of thumb, this can be calculated as N > 500 c/(a × t), where “c” represents the largest number of attribute levels for any one attribute, “a” represents the number of alternative scenarios, and “t” the number of choice tasks. For our experiment, this resulted in a required sample size of 107 participants [(500 × 3)/(2 × 7)]; however—for robust quantitative research where inferences are to be made to a larger population and where no subgroup analyses are planned—a minimum of 300 participants is recommended.^20,23^ Because this study was nested within a larger parent trial and timing and resources devoted to recruitment into the DCE were constrained by progress in the larger study, we sought to recruit a minimum of 300 and up to 500 patients on ART—if possible—to participate in the DCE.

### Data Collection

Research assistants who had been trained on the use of the device and administration of the questions collected data on computer tablets. The tool was translated into the 3 main local languages: Bemba, Nyanja, and Tonga, and research assistants read out each question and response level to participants in their local language. The DCE data were collected before any other general survey questions were undertaken to limit response fatigue. Participant demographic characteristics with regard to age, sex, marital status, educational level, employment status, and clinic setting were collected as part of the general survey. The standard operating procedures for data collection are available in Supplemental Digital Content 3, http://links.lww.com/QAI/B323.

### Statistical Analysis

Descriptive characteristics were tabulated, and characteristics of those who did and did not receive the DCE were compared. To evaluate the strength of patient preferences for DSD attributes, we used regression models to calculate preference weights (marginal utility or β-coefficient) and determined the mean relative preference for each level compared with a baseline attribute level—for example, the preference for collecting ART at a clinic was compared with the preference for collecting ART in the community. The β-coefficient in these models represents the relative strength of patient preferences for an attribute level compared with the reference level, with positive values representing a positive preference and negative values representing negative preferences, and higher β values representing stronger preferences. To account for heterogeneity of preferences across the population, we used mixed (random parameter) logit models. Mixed logit models present the relative mean preference weights (β-coefficients), as well as SDs of effects across the sample, and capture the heterogeneity across participants.^20,24^ All attributes were modeled as categorical variables, and we assumed an independent covariate matrix. Goodness of fit was determined by comparing models with different random parameters using the Akaike information criterion, Bayesian information criterion, and likelihood ratio tests {LR = 2 × [e(ll)_alt_ − e(ll)_null_]}. Based on the results of the likelihood ratio test, we selected a final model with random parameters for all attributes. In a trade-off analysis, we subtracted the absolute values of β-coefficients of all other significant attributes from the strongest preference weight resulting in a “β-difference,” to test what combination of attributes were equivalent to—and what patients would be willing trade for—their strongest preference. To explore preference heterogeneity, we evaluated the interaction between mean preferences and patient characteristics including age, sex, and health care setting in the model. We maintained only the interaction terms between health care setting and the location of ART pick-up, and health care setting and frequency of ART pick-up in the final model; these interactions seemed to explain substantial heterogeneity (*P* < 0.001). We further quantified preference heterogeneity within rural and urban health care settings by deriving preference probabilities from the parameters of the mixed logit regression.^25^ STATA Version 14 and R statistical software packages were used for all analyses.^26,27^

## RESULTS

### Patient Characteristics

Between July 12 and Dec 14, 2016, 1346 clinic attendees from 12 health care facilities across 3 provinces in Zambia were enrolled into the general survey; of these, 796 were on ART. A random sample of those on ART received the DCE (N = 486). The 486 ART patients who received the DCE were similar to the 310 who did not (Table 2). Most DCE participants were female (N = 288, 59%), married (N = 294, 60%), with a minimum of primary school education (N = 366, 75%) and living in an urban setting (411, 85%). The median age of participants was 39 years (IQR: 33–36) (Table 2). A total of 244 patients received the first block of 7 questions and 242 received the second block.

**TABLE 2.**
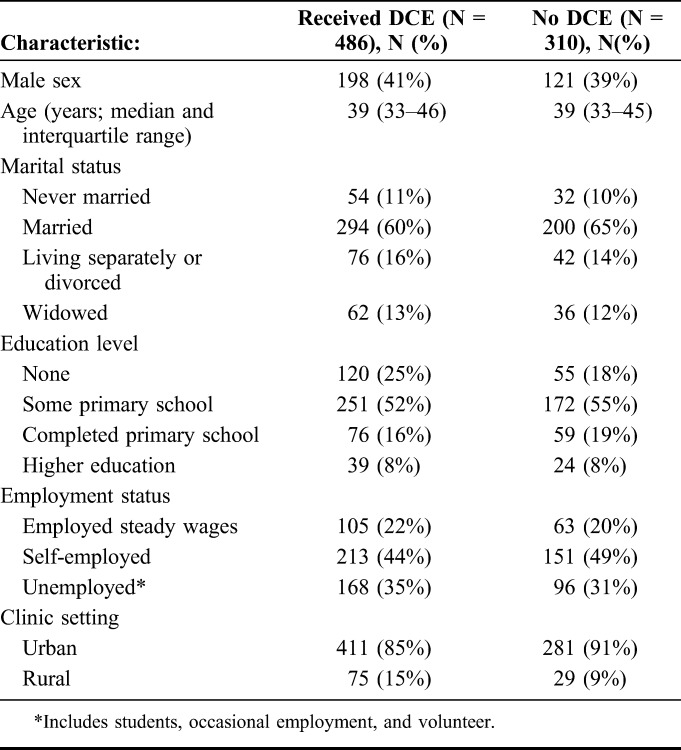
Baseline Characteristics of DCE Eligible Participants

### Stated Preferences for Differentiated Service Delivery

**FIGURE 1.**
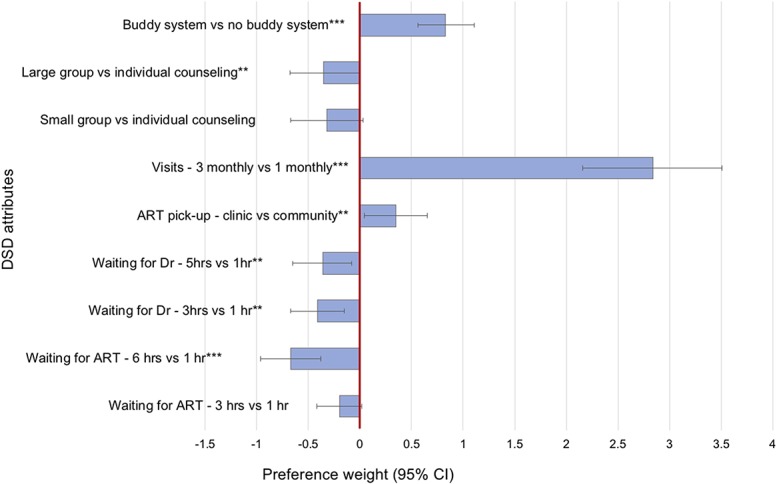
Mixed logit model—preferences for DSD attributes.

Results from the mixed logit model (Fig. 1 and Table 3) revealed that the strongest overall preference was for reducing clinic visit frequency, from 1 monthly to 3 monthly visits (preference weight: β = 2.84; 95% CI: 2.16 to 3.51; *P* < 0.001), patients preferred 3 monthly visits almost 3 times more than 1 monthly visits. Patients showed milder preferences for reducing time spent waiting at the clinic; they preferred waiting for ART for 1 hour as opposed to the longest waiting time of 6 hours (β = −0.67; 95% CI: −0.96 to −0.38; *P* < 0.001) but were less concerned about the shorter waiting time of 3 hours compared with 1 hour (β = −0.20; 95% CI: −0.42 to 0.02; *P* = 0.070) and similarly preferred waiting to see the doctor for 1 hour as opposed to 3 hours (β = −0.41; 95% CI: −0.67 to −0.15; *P* = 0.002) or 5 hours (β = −0.36; 95% CI: −0.65 to −0.07; *P* = 0.013). They also indicated some preference for having a “buddy” collect their ARVs for them at nonclinic visits as compared to always collecting ARVs themselves (β = 0.84; 95% CI: 0.56 to 1.11; *P* < 0.001) and collecting medications at the clinic as opposed to in the community (β = 0.35; 95% CI: 0.04 to 0.65; *P* = 0.027). There was also a slight preference for individual counseling compared with large group counseling (β = −0.35; 95% CI: −0.68 to −0.01; *P* = 0.041).

**TABLE 3.**
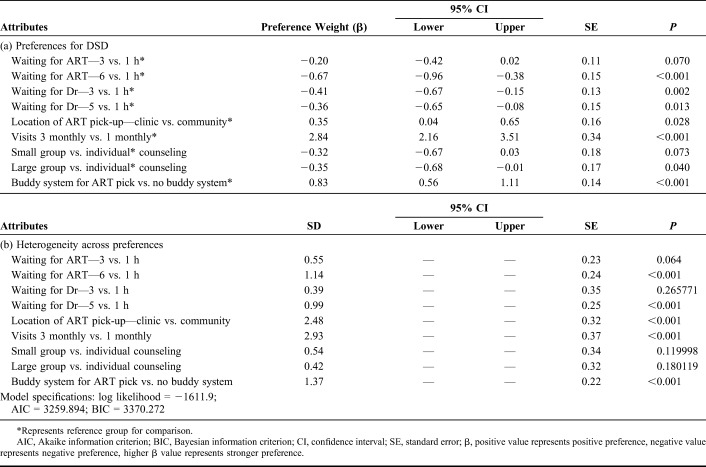
Mixed Logit Model—(a) Preferences and (b) Heterogeneity of Preferences for Differentiated Service Delivery Attributes

### Trade-off

In the assessment of trade-offs between service features, we determined that the preference for reducing visit frequency from 1 to 3 months was equivalent to the combination of all other significantly preferred service delivery features, suggesting that patients were willing to give up: 1 hour of doctor waiting time compared with 3 or 5 hours, buddy collection of ART versus no buddy system, individual instead of large group counseling, community ART pick-up instead of clinic, and 1 hour of pharmacy waiting time compared with 6 hours, to attend clinic every 3 months instead of 1 monthly (β difference = 0.23; 95% CI: −0.41 to 0.86; *P* = 0.487).

### Preference Heterogeneity

**FIGURE 2.**
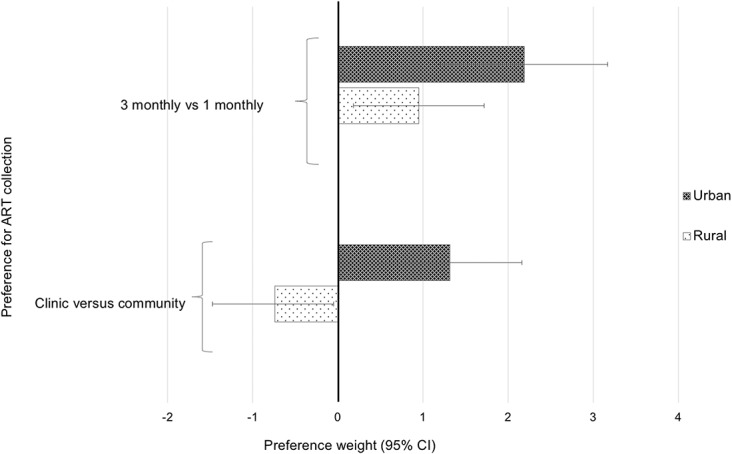
Heterogeneity of preferences for ART collection, by setting.

To evaluate whether patient preferences varied by key measurable characteristics, we entered covariates: age, sex, and facility setting into the mixed logit model. Substantial preference heterogeneity was demonstrated by setting (Fig. 2): there was greater preference among urban (compared with rural) patients for collecting ART in the clinic rather than in the community (urban-β = 1.32; 95% CI: 0.48 to 2.16; *P* < 0.001 vs. rural-β = −0.74; 95% CI: −1.47 to −0.01; *P* = 0.049) and for reducing frequency of ART pick-ups from 1 to 3 months (urban-β = 2.19; 95% CI: 1.20 to 3.17; *P* < 0.001 vs. rural-β = 0.95; 95% CI: 0.18 to 1.72; *P* = 0.015). There were no other interactions between differentiated care attributes and patient characteristics. The variation in preferences between rural and urban participants regarding location for ART collection and frequency of drug pick-up explained some of the heterogeneity detected in preferences; however, substantial preference heterogeneity remained across the population, even after accounting for setting as evidenced by persistent large and significant SDs in the mixed logit model for several service features (see Supplemental Digital Content 4, http://links.lww.com/QAI/B323). Preference heterogeneity also existed within setting subgroups (as determined through integral estimation), and although rural participants preferred community ART collection overall, approximately 40.1% still wanted to collect ART in the facility, and similarly among urban participants, most (67.3%)—but not all—preferred clinic to community ART collection.

## DISCUSSION

This study demonstrates that, in this patient population, the strongest overall preference is for reduced clinic visit frequency, and that between patient subgroups and individuals, there is substantial variation in preference. Although other features such as buddy ART collection systems, and reduced waiting time at the health facility were also desired, it appeared—particularly among urban patients—that reducing the number of clinic visits was the most valued differentiated service feature. Patients also showed an overall preference for collecting ART at the health facility instead of in the community—but on stratification by location of residence, we found a strong preference for getting treatment at a facility among urban participants while rural patients showed, on average, a mild preference for community ART collection. The differences between rural and urban participants regarding location of ART collection accounted for some of the statistical heterogeneity in preferences; however, preference heterogeneity was still evident within the rural and urban subgroups—evidenced by large and significant SDs for several of the estimated preference weights: even among rural patients who as a group would like to receive care in the community, some still prefer to get care at the facility. This heterogeneity has been identified in other cohorts in South Africa^28^ and highlights the fact that in settings where community-based treatment is being considered by health authorities, patients may still be given the default option of being treated in the facility—an option that has minimal costs for a health system.

To date, several DSD models have been shown to be effective in retaining patients in care, and their features are generally acceptable to patients in the short to medium term.^4,7–11,29^ Scale-up, however, increases complexity, and the choice of model, design, resource requirements, method of implementation, and monitoring is highly context-specific.^3,30^ As a result, national health ministries will have to decide which models to prioritize. This experiment provides evidence of relative patient preferences for DSD model features that are relevant to the Zambian context and can be used in DSD model selection for both rural and urban patients.

These findings suggest prioritizing and strengthening systems, which support individual or health worker-managed models,^1^ which are facility-based and focus on multimonth prescribing in urban areas, because they are relatively easy to implement by the health system and also conform broadly to patient desires. For those in rural areas, health worker-managed groups,^1^ client managed groups, or out-of-facility individual models which in addition to distributing ART in the community allow for reduced ART collection frequency should also be considered. Although broad public health approaches are needed to expand access to ART, some element of patient choice should also be incorporated into model design where possible.

This study had a number of limitations. First, the design of the experiment was not orthogonal or balanced which are common design features of such experiments, where all attributes and all attribute pairs appear equally across the experiment; however, lack of orthogonality does not preclude parameter estimation, and near orthogonal designs such as this one can still produce reliable preference estimates.^20^ In our case, a rigorous approach was taken to design a statistical and response efficient experiment with the given number of attributes and levels specified. Second, most patients in this study were from an urban setting with only 15% from rural health centers; it is possible that the full breadth of preferences of rural participants were not represented in our data. Third, this group of patients had never been exposed to several of the differentiated service features that were presented to them in the study—such as buddy pick-up systems or community ART distribution—it is possible that some patients could struggle to determine their preference for care which they had never experienced. Finally, the findings from this experiment may not be directly generalizable to settings outside of Zambia; however, our evidence of marked preference heterogeneity across a population and the use a DCE to identify key features of differentiated services can be translated to any setting.

Given the substantial preference for multimonth prescriptions for all patient subgroups in this study and the persistent desire for facility-based care—particularly in urban areas—settings such as Zambia could consider prioritizing implementation of multimonth prescriptions and facility-based care in urban centers. If additional community-based approaches are to be added for rural patients, preserving the ability to “opt out” of community-based models (in favor of facility-based care) would seem to allow systems to align best with demand and preference heterogeneity, as measured in this study.
